# Food safety assessments of acrylamide formation and characterizations of flaky rolls enriched with black rice (*Oryza sativa*)

**DOI:** 10.3389/fnut.2022.1027800

**Published:** 2022-10-21

**Authors:** Wen-Chien Lu, Yu-Tsung Cheng, Yung-Jia Chan, Po-Hsien Li

**Affiliations:** ^1^Department of Food and Beverage Management, Chung-Jen Junior College of Nursing, Health Sciences and Management, Chiayi City, Taiwan; ^2^Cardiovascular Center, Taichung Veterans General Hospital, Taichung, Taiwan; ^3^College of Biotechnology and Bioresources, Da-Yeh University, Changhua, Taiwan; ^4^Department of Food and Nutrition, Providence University, Taichung, Taiwan

**Keywords:** *Oryza sativa*, black rice, flaky rolls, acrylamide, furfurals, antioxidant

## Abstract

This study aims to investigate the physicochemical composition, textural parameters, and chemical constituent of flaky rolls incorporated with different proportions of black rice flour. According to farinographic characteristics, the addition of black rice flour could reduce the stability and increase the dough development time and water absorption (%). While for the extensographic properties, addition of black rice flour resulted in significantly different maximum resistance to extension (BU) and extensibility (cm) vs. the control. With the addition of black rice flour in flaky rolls, the crude protein, total dietary fiber (TDF), soluble dietary fiber (SDF), and insoluble dietary fiber (IDF) were significantly improved. Glucose released was much lower with 10 and 20% black rice than the control and 5% black rice because of the higher black rice inclusion. With increasing black rice incorporation, total anthocyanin content, and antioxidant capacity was also improved. The content of asparagine, acrylamide, hydroxymethylfurfural (HMF), furfural, methylglyoxal, and glyoxal in flaky rolls was also increased. The proper content of black rice flour (5%) could significantly enhance the stability of the dough properties; control the final volume, texture, and appearance; and retain good protein and fiber composition, antioxidant capacity, and overall acceptance of the flaky roll.

## Introduction

Rice (*Oryza sativa*) is one of the top three most vital kinds of cereal and essential food for over half of the world's population (1). This cereal has an extensive genetic diversity, with thousands of varieties grown worldwide. Of the pigmented rice varieties, black rice (*Oryza sativa* L.) has gained attention because of its sensory characteristics, beneficial nutritional value, and also functional health attributes (2). Black rice is not just a good source of carbohydrates and fiber, but also contains abundant nutrition and bioactive constituents, such as essential amino acids, functional lipids, dietary fiber, vitamins (B complex, A, and E), minerals (K, Fe, Zn, Cu, Mg, Mn, and P), anthocyanins, phenolic compounds, and phytosterols (3–6). Moreover, black rice grain has been recognized as a diabetes controller in Chinese folk medicine, even though the white rice was a main glycemic load contributor in many rice-consuming communities (7, 8).

Previous epidemiological studies presented that black rice had demonstrated plentiful health-promoting qualities, for example, the high antioxidant activity, endothelial cells protection, cardiovascular disease prevention, lipid peroxidation inhibition, immunomodulation, and anti-tubercular, anti-ulcer, anti-inflammatory, and potent anticancer activity (9–11). A previous study found that black rice act as extraordinary raw ingredients (functional properties) in food products because the roasting process may enhance the phenolic contents and α-glucosidase inhibition activity (8). However, the formation of contaminants, such as acrylamide, hydroxymethylfurfural (HMF), or furfural of baking products substituted with black rice, is rarely discussed. The reaction between asparagine (free amino acid) and reducing sugar (for example glucose and fructose) to generate acrylamide was called Maillard reaction (10). Acrylamide was classified as a grade of Group 2A, which means that it is probably carcinogenic to humans. Furthermore, a previous study concluded that higher dose of acrylamide was carcinogenic, genotoxic, and neurotoxic tested organisms (11, 12).

Flaky rolls have the mutual name Phoenix egg rolls in Macau and egg biscuits in Southeast Asia countries; they are known as short dough biscuits in Taiwan (13). Flaky rolls are produced by using multiple fold-in methods. First, a thin layer of flour batter is spread on a hot circular metal pan, then the cooked batter is folded one-quarter on each side and rolled around a flat rod to produce a flaky roll.

This work builds upon a previous study showing that rice protein hydrolysates as an egg replacement effectively sustained the functional and physicochemical properties along with flavor and texture of flaky rolls and possibly be an egg replacement (13). To the best of our knowledge, no scientific report has described the impact of processing on the incorporation of flaky rolls with different proportions of black rice flour. Thus, this current research aimed to study the physicochemical properties, textural characteristics, *in vitro* starch digestibility, antioxidant capacity, and acrylamide formation of flaky rolls incorporated with different proportions of black rice flour. Also, the pasting, farinographic, and extensographic properties of the flour mixture were analyzed to further understand the characteristics of the dough and its mechanical strength during processing. We provide help in achieving optimal black rice flour formulations to obtain good-quality final products.

## Results and discussions

### Rheological characteristics and pasting properties of dough

The rheological characteristics and pasting properties of wheat dough with different proportions of black rice flour are in Table 1. Farinograph characteristics of dough are studied to determine the quality of the flour and are assessed with a farinograph (14). The farinograph determines the characteristics of the dough by measuring and recording the mechanical strength of the dough during processing, which play a vital role to obtain optimal flour mixtures of good quality products. The water absorption (%) of the dough, which is the amount of water required by the dough to reach the standard consistency of 500 BU, was increased from 63.6 ± 0.4 to 67.7 ± 0.2 as the content of black rice flour increased from 0 to 20%. The dough development time (time for the dough to form and achieve consistency of 500 BU) was increased from 1.9 ± 0.4 min (control) to 2.7 ± 0.1 min (20 BR), with no significant difference between 10 BR (2.8 ± 0.2 min) and 20 BR. The breakdown time (min), stability (min), and peak time (min) were reduced gradually from the control to 20 BR sample. The higher content of black rice flour caused the lower amount of time (min) in which dough kept its standard consistency with the kneading process (stability time). Farinograph stability time is correlated with flour strength. Short stability times are generally more suitable for a variety of biscuits (flaky rolls) and often require shorter mixing times (15). Significant correlations have been found between stability time and volume of the flaky roll. The higher the stability time (Table 1), the larger the volume of the flaky roll (Table 2). In short, adding black rice flour verified significant changes in dough farinograph parameters: water absorption and dough development time increased and breakdown time, peak time, and stability of the dough decreased.

**Table 1:** Rheological characteristics and pasting properties of wheat dough with different proportions of black rice flour.

**Treatments**	**Control**	**5 BR**	**10 BR**	**20 BR**
**Farinographic characteristics**
Water absorption (%)	63.6^d^±0.4	65.7^bc^±0.7	66.4^b^±0.3	67.7^a^±0.2
Dough development time (min)	1.9^c^±0.4	2.4^b^±0.1	2.8^a^±0.2	2.7^a^±0.1
Breakdown time (min)	24.5^a^±0.2	14.7^b^±0.4	12.3^c^±0.1	11.9^d^±0.6
Stability (min)	21.7^a^±0.7	17.4^b^±0.5	11.7^c^±0.1	9.9^d^±0.6
Peak time (min)	10.7^a^±0.5	7.7^b^±0.1	6.9^c^±0.2	5.7^d^±0.2
**Extensographic properties**
**Maximum resistance to extension (BU)**	
45 min	598^d^±11.3	701^c^±10.5	797^b^±9.2	892^a^±13.1
90 min	731^c^±17.2	914^b^±7.1	919^b^±6.7	967^a^±15.2
135 min	733^d^±10.5	856^c^±8.2	982^b^±5.4	1089^a^±16.8
**Extensibility (cm)**	
45 min	17.4^a^±0.6	14.5^b^±2.9	13.5^c^±4.2	12.7^d^±1.1
90 min	12.6^a^±1.3	11.4^b^±1.3	10.2^c^±3.7	9.3^c^±1.3
135 min	14.8^a^±2.4	10.7^b^±0.7	10.3^b^±2.5	8.7^c^±0.5
**Pasting parameters**
Peak viscosity (BU)	860.7^a^±6.9	838.5^b^±7.8	815.2^c^±2.7	734.4^d^±6.3
Breakdown (BU)	184.2^a^±6.1	147.2^b^±1.4	144.9^b^±3.6	87.4^c^±3.4
Setback (BU)	517.5^a^±4.2	488.5^b^±1.6	480.3^b^±5.2	478.6^c^±3.7
Final viscosity (BU)	1178.3^a^±5.1	1157.4^b^±6.7	1169.3^b^±3.5	1124.7^c^±7.2

^1^Formulations for 5, 10, and 20 BR are 5, 10, and 20% incorporated black rice.

^2^Data are mean± SD.

^3^Different letters significantly represent difference (P < 0.05) from the control.

**Table 2:** Characteristics and textural properties of flaky rolls incorporated with different proportions of black rice flour.

**Properties**	**Control**	**5 BR**	**10 BR**	**20 BR**
**Characteristic properties**
Length (cm)	6.62^a^±0.33	6.54^b^±0.13	6.47^b^±0.27	6.32^c^±0.07
Width (cm)	3.96^a^±0.19	3.88^a^±0.11	3.78^a^±0.08	3.67^b^±0.12
Thickness (cm)	1.44^a^±0.22	1.41^a^±0.13	1.35^a^±0.08	1.33^a^±0.15
**Textural properties**
Hardness (N)	27.43^b^±1.41	28.52^a^±1.58	28.77^a^±1.15	29.41^a^±2.01
Fracturability (mm)	6.27^a^±0.19	5.97^b^±0.18	5.84^b^±23	5.72^b^±0.33
Springiness (mm)	0.77^a^±0.03	0.64^b^±0.07	0.61^b^±0.09	0.57^b^±0.06
Cohesiveness	0.24^c^±0.06	0.26^b^±0.04	0.27^b^±0.02	0.31^a^±0.03
Adhesiveness (N.S)	48.82^a^±3.57	41.61^b^±4.29	40.29^b^±6.31	36.71^b^±6.23
**Color properties**
L*(lightness)	70.14^a^±6.57	61.52^ab^±5.43	60.78^b^±4.33	56.43^c^±3.97
a*(redness-greenness)	12.57^b^±0.35	12.57^b^±0.29	13.07^b^±0.31	14.15^a^±0.39
b*(yellowness-blueness)	30.54^a^±1.29	30.54^a^±1.14	29.93^a^±2.13	26.57^b^±3.11

^1^Formulations for 5, 10, and 20 BR are 5%, 10%, and 20% incorporated black rice.

^2^Data are mean± SD.

^3^Different letters significantly represent difference (P < 0.05) from the control.

The maximum resistance to extension (BU) in the extensographic properties of the dough showed an increasing trend, whereas the extensibility (cm) showed a decreasing trend. The pasting properties of formulated flour were examined with the Rapid Visco Analyzer, a rotational or stirring rheometer that measures the apparent viscosity of starch-containing suspensions under variable and controlled heating (cooking), cooling, and shear stress conditions. The rheological measurement is crucial to characterize the material's performance during processing and to attempt to predict final product quality (16). The addition of 20 BR had the lowest pasting viscosity with the lowest peak viscosity (734.4 ± 6.3 BU), breakdown viscosity (87.4 ± 3.4 BU), setback viscosity (478.6 ± 3.7 BU), and final viscosity (1124.7 ± 7.2 BU) as compared with 5 and 10 BR. The peak viscosity (highest viscosity reached during pasting) and final viscosity (viscosity at the end of the pasting cycle) with 5 BR were 838.5 ± 7.8 BU and 1157.4 ± 6.7 BU, respectively. The peak viscosity of the different flour formulations significantly differed. The peak viscosity of composite flour decreased with increasing black rice flour content, perhaps because of the higher content of protein and fiber in flaky rolls (Table 3), which has been reported to lower viscosity with increasing black rice flour content (17). Amylose content and long chain fraction of amylopectin have been reported as major factors influencing the peak viscosity (18). Furthermore, Amylose and amylopectin content, and hydrophobic interaction between the amylose was considered as one of the major factors that will affect the pasting, and thermal property of starches (19). The results correspond to a previous study that evaluated wheat flour biscuits incorporated with okra flour (17).

**Table 3:** Nutrition composition (on a dry weight basis) of flaky rolls incorporated with different proportions of black rice flour.

**Compositions**	**Control**	**5 BR**	**10 BR**	**20 BR**
Moisture (g/100 g)	3.17^a^±0.06	3.06^b^±0.04	2.94^c^±0.02	2.73^c^±0.03
Total starch (g/100 g)	67.21^a^±1.43	59.41^b^±2.74	56.52^b^±1.45	50.41^c^±2.19
Crude protein (g/100 g)	18.82^b^±0.33	19.18^b^±0.25	20.52^b^±0.17	22.14^a^±0.39
Crude lipid (g/100 g)	10.25^b^±0.27	11.53^a^±0.15	11.77^a^±0.33	11.93^a^±0.36
TDF (g/100 g)	4.12^d^±0.07	5.03^c^±0.11	5.74^b^±0.15	6.42^a^±0.03
SDF (g/100 g)	0.73^c^±0.02	1.24^b^±0.04	1.39^b^±0.07	1.62^a^±0.05
IDF (g/100 g)	3.27^c^±0.17	4.18^b^±0.43	4.52^a^±0.36	5.02^a^±0.48
Ash (g/100 g)	1.68^b^±0.02	1.71^b^±0.09	1.78^b^±0.02	1.96^a^±0.07
Free sugar (g/100 g)	3.62^a^±0.23	3.71^a^±0.19	3.77^a^±0.26	3.82^a^±0.17
Cholesterol (mg/100 g)	0.62^a^±0.03	0.69^a^±0.05	0.66^a^±0.01	0.68^a^±0.04

^1^Formulations for 5, 10, and 20 BR are 5%, 10%, and 20% incorporated black rice.

^2^Data are mean± SD.

^3^Different letters significantly represent difference (P < 0.05) from the control.

TDF, total dietary fiber; SDF, soluble dietary fiber; IDF, insoluble dietary fiber.

### Nutritional composition analysis

Table 3 shows the nutritional composition of flaky rolls with varying amounts of black rice flour. The moisture content of the flaky rolls decreased with increasing black rice flour content, with values from 2.73 to 3.17%. Controlling moisture content is vital due to the high moisture content is not desirable in crispy and crunchy products such as flaky rolls because of the inverse relationship between the texture of the products and the possibility of microbial attack. The crude protein increased with black rice amount, with 20 BR having the highest value (22.14 ± 0.39 g/100 g) and the control sample having the least value (18.82 ± 0.33 g/100 g). Rice contains a more completed and balanced amino acid composition than wheat and corn because of its higher lysine and Sulfur-containing amino acid content (20). Thus, in black rice and wheat composite flaky rolls, the composite flour can serve as a source of protein and solve the problem of protein-energy malnutrition. With increasing black rice content, the ash content also increased, with no substantial difference for free sugar or cholesterol content. The fiber content of the flaky rolls increased with increasing black rice flour content, with values from 4.12 to 6.24 g/100 g (TDF), 0.73 to 1.62 g/100 g (SDF), and 3.27 to 5.02 g/100 g (IDF). The result presented that an inversed relationship between both compounds was found as the total starch (g/100 g) decreased, free sugar content (g/100 g) raised up. Complicated chemical reactions, for example the Maillard reaction and caramelization take place during the baking process of flaky rolls, moreover, these reactions involved glucose and fructose, which generated from starch and sucrose hydrolysis during baking. Therefore, the total starch and free sugar content was analyzed.

### Dimensional quality and textural analysis

**Figure 1:**
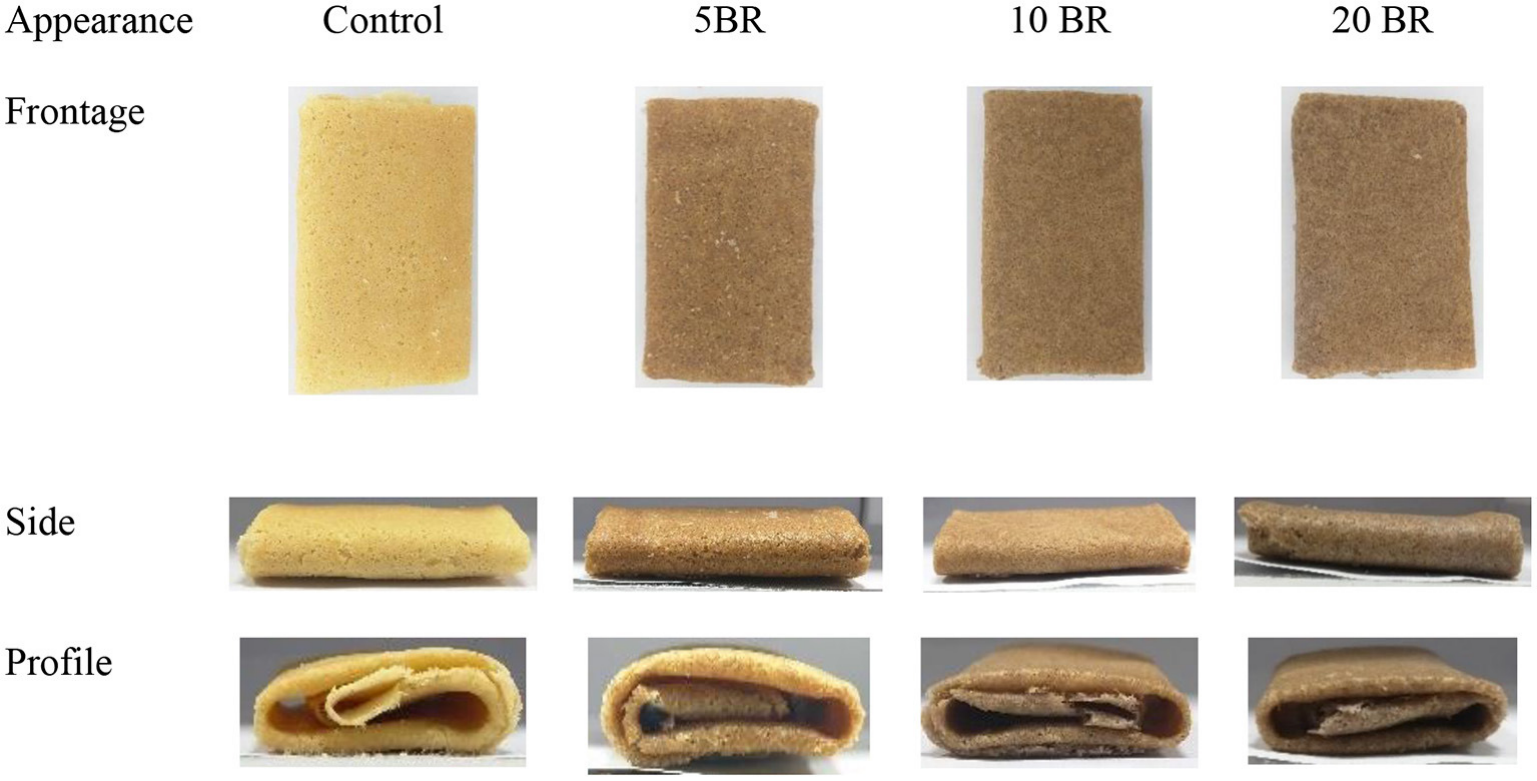
The appearance of flaky rolls incorporated with black rice flour at different proportions.

**Figure 2:**
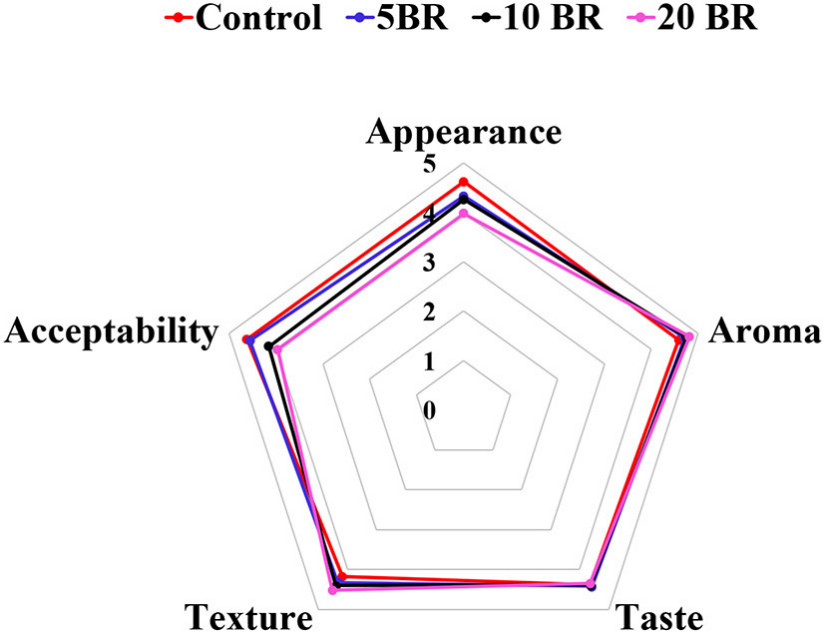
Sensory evaluation of flaky rolls incorporated with different amounts of black rice flour.

The flaky roll images of flaky rolls incorporated with different amounts of black rice flour are in Figure 1, and the textural properties and color changes are in Table 2. The hardness, fracturability, springiness, and adhesiveness of the flaky roll did not significantly differ among 5, 10, and 20 BR. However, the textural properties of the control sample significantly differed with black rice flour incorporation. The results agreed with the sensory evaluation results (Figure 2), showing that the texture attributes greatly differed from the control sample (4.17 ± 0.18), with no significant difference between 5 BR (4.33 ± 0.19) and 10 BR (4.40 ± 0.21), and only slightly different from 20 BR (4.52 ± 0.13). These results indicate that the incorporation of black rice flour affected the texture of the flaky roll. High dietary fiber content was competing to absorb the water and enfolded the starch granules dispersed in the gluten network structure, which prohibited the protein molecules from being crisscrossed strongly and affected the formation of a spatial network (21). The hardness of the flaky rolls was determined by the composite matrix of protein aggregates, lipids, and sugars embedded within ungelatinized starch granules. If the hardness of the flaky roll is too high, it will affect the taste and cause the biscuits to be not crispy enough. However, if the hardness of the flaky roll is too low, the flaky roll will be broken easily. Cohesiveness discussed the quantity of internal binding force needed to form a sample, it reflects the power of the interaction between molecules within the sample or between various structural elements. Meanwhile, it also represents the capability of the sample to resist damage and maintain its integrity. With the fortification of black rice flour, the cohesiveness of the flaky rolls was increased, resulting in the flaky roll having suitable crispiness, not being easily broken, and being easily transported and stored.

**Figure 3:**
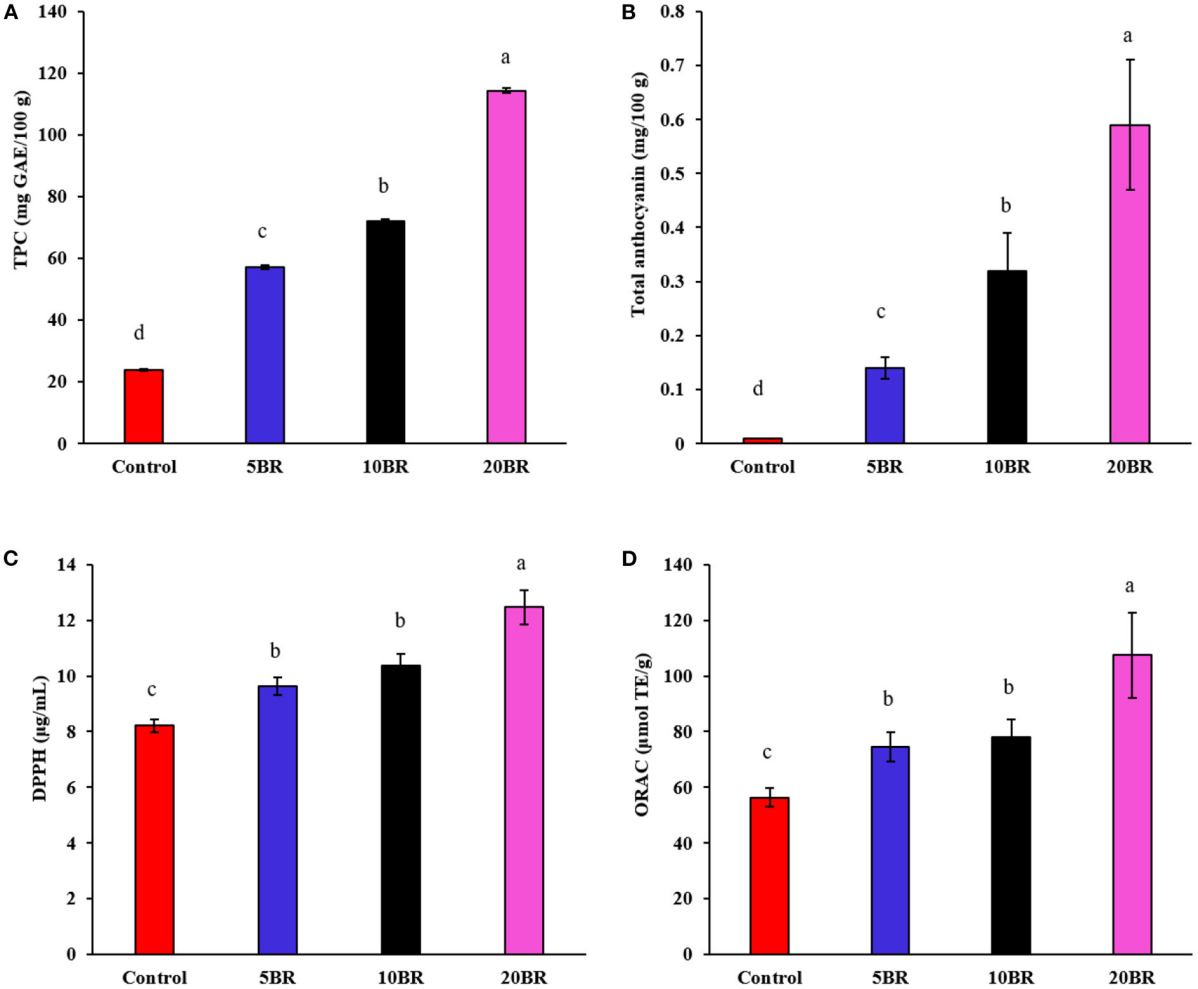
The *in vitro* antioxidant capacity of flaky rolls incorporated with different proportions of black rice flour: **(A)** total phenolic content; **(B)** total anthocyanin; **(C)** DPPH scavenging activities; and **(D)** ORAC assays. Different letters significantly represent difference (*P* < 0.05) from the control.

Evaluation of color attributes was vital because the first sight appearance may affect the consumer's acceptability. L^*^ values decreased from the control (70.14 ± 6.57) to 20 BR (56.43 ± 3.97), and b^*^ values (yellowness-blueness) also decreased from 30.54 ± 1.14 (5 BR) to 26.57 ± 3.11 (20 BR) with increasing black rice flour content (Table 2). In contrast, a^*^ values (redness-greenness) increased steadily from the control sample to 20 BR, from 12.57 ± 0.35 to 14.15 ± 0.39. Color development in biscuits is primarily depending on the Maillard reaction and caramelization occurring during the baking process. As well, the dextrinization of starch present in wheat flour may be liable for color development. Thus, the results of black rice flour incorporated flaky rolls agreed with the study of Mieszkowska and Marzec (22), finding that color was greatly affected by polydextrose and protein-rich ingredients, due to the Maillard reaction progression and leading to a decline in lightness (L^*^ values) and raised in redness (a^*^ values). Meanwhile, anthocyanins contents increased with an increasing proportion of black rice (Figure 3), which might result in the purple-color appearance of the flaky rolls.

### Sensory evaluation

The mean value of sensory ratings for flaky rolls incorporated with different proportions of black rice flour is in Figure 2. Sensory characteristics, for instance, the appearance, aroma, taste, texture, and overall acceptability were evaluated. The scores for 5 and 10 BR did not significantly differ in the sensory properties of appearance, aroma, taste, and texture, except for overall acceptability. The mean aroma score did not greatly differ for 5 BR (4.72 ± 0.14), 10 BR (4.75 ± 0.11), and 20 BR (4.81 ± 0.23). However, the aroma differed between all flaky rolls incorporated with black rice flour proportions and the control (4.58 ± 0.13). In general, the incorporation of black rice flour increased the aroma and texture of the flaky rolls, not so much the taste, and affected the appearance and overall acceptability. Hence, suitable incorporation amount of black rice flour should be concerned for the further product development and marketing.

### Total phenolic content, total anthocyanin content, and *in vitro* antioxidant capacity

Figure 3 presents the TPC, total anthocyanin content, and *in vitro* antioxidant assay results (DPPH and ORAC) for flaky rolls incorporated with different proportions of black rice flour. TPC, DPPH, and ORAC for 5, 10, and 20 BR increased markedly from 57.14 ± 0.65 to 114.38 ± 0.92 mg GAE/100 g, 9.64 ± 0.31 to 12.47 ± 0.61 μg/mL, and 74.62 ± 5.21 to 107.43 ± 15.33 μmol TE/g, respectively. With increasing black rice flour content, total anthocyanin content increased from 0.14 ± 0.02 (5 BR) to 0.32 ± 0.07 (10 BR), and 0.59 ± 0.12 (20 BR), causing increased TPC, DPPH, and ORAC. Black rice contains a high content of phenolic acids, commonly characterized as free or bound phenolic acids (9). The dark pigment color of black rice is principally due to the high content of anthocyanin, a group of natural reddish to dark-purple colors of flavonoids (20). A previous study identified that anthocyanins in black rice extract (containing 41.69% of anthocyanins) showed brilliant *in vivo* antioxidant activity, as indicated by the model of KBrO_3_-induced renal injury in mice; the protective effects were due to the free radical scavenging capacity of the anthocyanins (23). Furthermore, anthocyanins and other bioactive compounds of interest in black rice showed potent anti-inflammatory effects both *in vitro* and *in vivo* by regulating NF-κB and MAPK (24).

### Starch digestion

**Figure 4:**
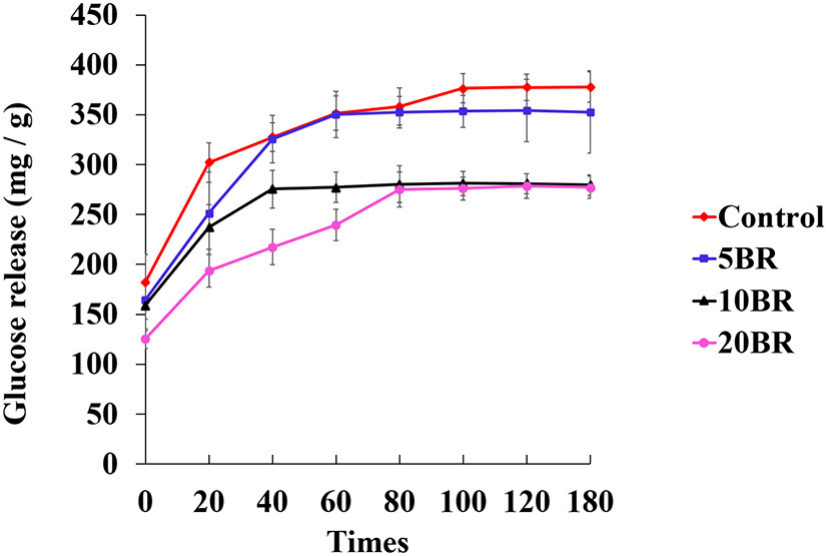
*In vitro* starch digestion at different times for flaky rolls incorporated with different proportions of black rice flour.

Figure 4 shows the *in vitro* starch digestion of flaky rolls incorporated with different proportions of black rice flour. Glucose release is measured as the reducing sugars degraded from starch by digestive enzymes. Starch hydrolysis gradually increased from 182.47 ± 27.58 (0 min) to 377.79 ± 15.11 (180 min) mg/g, for the control sample. Likely, the maximum glucose concentration was in the control sample. However, glucose release was downregulated for all flaky rolls with black rice flour. The glucose released for 5 BR increased steadily for 120 min (354.21 ± 31.19 mg/g) and decreased at 180 min (352.57 ± 41.28 mg/g). For 10 BR, the glucose released slowly increased from 159.14 ± 25.15 mg/g (0 min) and peaked at 100 min (281.17 ± 12.23 mg/g), then slightly decreased to 280.93 ± 9.88 mg/g at 120 min and 279.57 ± 10.29 mg/g at 180 min. For 20 BR, glucose release was only slightly increased from 125.71 ± 9.82 mg/g (0 min) to 278.42 ± 12.23 mg/g (120 min), then decreased to 277.45 ± 11.23 mg/g at 180 min. The glucose release was much lower for 10 and 20 BR than for the control and 5 BR. Dietary fiber influences digestion by altering the physical and chemical properties of the starch system (21). In addition, dietary fiber can increase the matrix viscosity at a gastrointestinal level, which is also related to the formation of a gel. Furthermore, dietary fiber may enclose the starch grains and prevent the amylolytic activity of digestive enzymes, hence interrupting the release of free glucose and resulting in a lower glycemic response or glucose released (25). Thus, the incorporation of black rice flour might restrict the *in vitro* digestion and hydrolysis of starch to a certain extent, and the glucose released was decreased with an increasing proportion of black rice flour.

### Free asparagine, acrylamide, HMF, furfural, methylglyoxal, and glyoxal in flaky rolls

**Figure 5:**
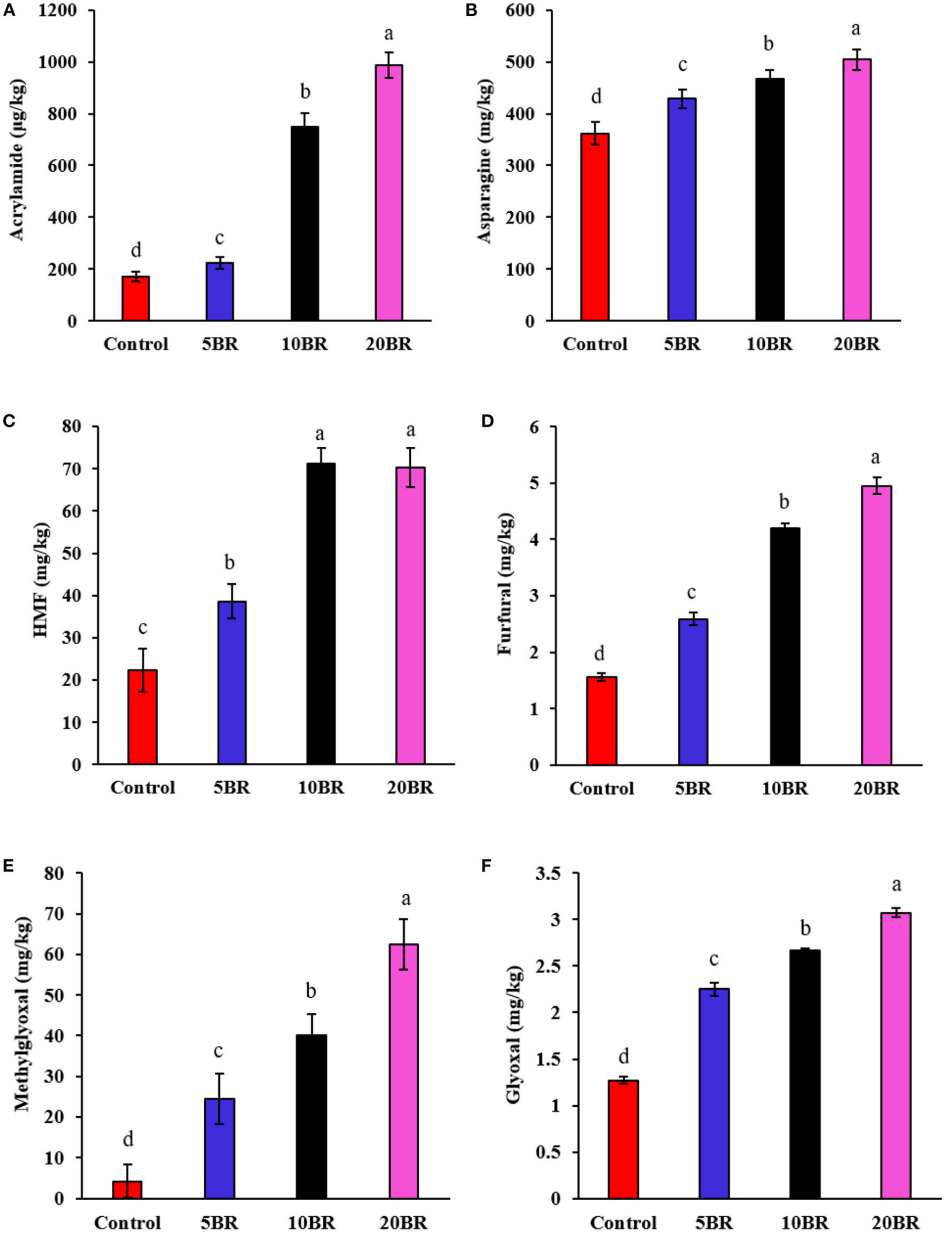
Content of asparagine, acrylamide, HMF, furfural, methylglyoxal, and glyoxal in flaky rolls incorporated with different proportions of black rice flour: **(A)** acrylamide content; **(B)** asparagine content; **(C)** HMF content; **(D)** furfural content; **(E)** methylglyoxal (MGO) content; and **(F)** glyoxal (GO) content. Different letters significantly represent difference (*P* < 0.05) from the control.

The content of asparagine, acrylamide, HMF, furfural, methylglyoxal, and glyoxal in flaky rolls incorporated with different proportions of black rice flour is in Figure 5. The acrylamide content increased sharply from 222.59 ± 22.41 μg/kg (5 BR) to 748.57 ± 52.15 μg/kg (10 BR) and 986.54 ± 49.13 μg/kg (20 BR) with an increasing proportion of black rice flour (Figure 5A). Meanwhile, acrylamide precursors, asparagine content, was also increased from 428.57 ± 17.38 mg/kg (5 BR) to 467.49 ± 16.52 mg/kg (10 BR) and 504.25 ± 19.28 mg/kg (20 BR) as compared with the control sample (362.17 ± 22.15 mg/kg) (Figure 5B). The European Food Safety Authority (EFSA) has confirmed that acrylamide increases the risk of cancer in all age groups, meanwhile ESFA also established a maximum allowable limit for acrylamide in food, and it has called for “indicative values” for acrylamide to be as low as possible (26). The acrylamide content of the control sample (171.15 ± 19.52 μg/kg) and 5 BR (222.59 ± 22.41 μg/kg) was lower than the benchmark level for “biscuits and wafers” (350 μg/kg) according to European Union regulations (27). Asparagine is an important precursor for acrylamide in cereal products. The content of free asparagine was determined in 11 milling fractions from wheat and rye. The higher the asparagine content, the higher the amounts of acrylamide formed in flaky rolls with black rice flour. The content of asparagine differs among along with within cereal species (28). Hence, selecting cereal species that consist of low levels of acrylamide precursors, which is the primary asparagine, is one of the food industry methods to reduce the acrylamide content in the final product (29).

HMF content rapidly increased from 38.59 ± 4.09 mg/kg (5 BR) to 71.15 ± 3.82 mg/kg (10 BR), and 70.25 ± 4.55 mg/kg (20 BR) with increasing proportion of black rice flour (Figure 5C). Also, furaldehyde (furfural) content increase with 5 BR (2.59 ± 0.11 mg/kg), 10 BR (4.19 ± 0.09 mg/kg), and 20 BR (4.95 ± 0.15 mg/kg) as compared with the control (1.56 ± 0.07 mg/kg) (Figure 5D). Complex chemical reactions such as the Maillard reaction and caramelization take place during the baking process of flaky rolls (30). Both reactions involve glucose and fructose, generated from starch and sucrose hydrolysis during baking. Among the many products formed, HMF, a possible mutagen, seems interesting because of its rapid accumulation during the process. HMF is not present in fresh and untreated foods but quickly accumulates during heat treatment and storage of carbohydrate-rich products (31). HMF is considered a good indicator of quality deterioration due to excessive heating or storage for a wide range of carbohydrate-containing foods (31) and is also used for inspecting the heating process of cereal products such as cookies or bread (32).

With an increasing proportion of black rice flour, the content of MGO, and GO was increased from 24.53 ± 6.22 to 62.52 ± 6.21 mg/kg and 2.25 ± 0.07 to 3.07 ± 0.05 mg/kg with 5 BR to 20 BR, respectively (Figures 5E,F). MGO is a reactive carbonyl compound commonly existing in thermally processed foods; it results from sugar degradation and lipid oxidation. MGO can yield numerous aroma compounds by reacting with amino acids and acts as a precursor to generating toxins, such as advanced glycation end products, acrylamide, and 4(5)-methylimidazole. Because of their highly nucleophilic reactivity with MGO, phenolic compounds capably scavenge MGO, thereby forming numerous adducts (33). The selective incorporation of compounds rich with amino acids or phenolic content may be a strategy to control MGO in foods.

## Conclusion

The substitution of black rice into flaky roll batters leads to nutritional improvement products, with lower total starch, higher protein content, and higher dietary fiber than the control. The incorporation of black rice flour significantly enhanced TPC, total anthocyanin content, and antioxidant capacity (DPPH and ORAC) and lowered the *in vitro* starch digestibility rate. Nevertheless, the fortification of black rice dramatically upsurges the formation of acrylamide, HMF, furfural, and MGO. Substitution of 10% black rice exceeded the acceptable value for acrylamide in flaky rolls. Thus, although the nutritional properties of flaky rolls were enhanced by the substitution of black rice, some risks correlated to the generation of contaminants after baking should be considered when designing new black-rice flaky-roll formulations.

## Materials and methods

### Materials

Black rice (*Oryza sativa* L.), subspecies *indica* black rice variety (Tainung Sen Glutinous 24) was cultivated from a local farm in Tianzhong Township, Changhua County, Taiwan. The black rice was milled and passed through a 100-μm sieve and stored in an air-tight aluminum container before further analysis. The analysis of moisture (Association of Official Agricultural [AOAC] Official Method 925.10), crude protein (AOAC Official Method AOAC 920.87), and ash content (AOAC Official Method AOAC 923.03) of the flour followed the methods of the AOAC (34). The moisture, crude protein, and ash content of black rice flour was 12.48, 9.82, and 1.84%, respectively.

Vedan Enterprise (Taichung, Taiwan) provided the rice protein powder (67% of protein, dry basis) with a moisture content of 4.75%, fat content of 8.63%, and carbohydrate content of 7.0%, respectively. The other basic baking ingredients, such as wheat flour (Sunright Foods Corp., Taipei, Taiwan; year of production: 2021; 1 kg per pack), margarine (Fareast Oil Corp., Taoyuan, Taiwan; year of production: 2021; 400 g per can), sugar powder (Taiwan Sugar Corp., Tainan, Taiwan; year of production: 2021; 1 kg per pack), cornflour (Sunright Foods Corp., Taipei, Taiwan; year of production: 2021; 1 kg per pack), lecithin (Taiwan Sugar Corp., Tainan, Taiwan; year of production: 2021; 200 g per can), and salt (Taiyen Biotech Co.,LTD. Tainan, Taiwan; year of production: 2021; 1 kg per pack), were purchased from Carrefour in Yuanlian City, Changhua Country, Taiwan. All chemicals used in this study were American Chemical Society (ACS) certified of analytical grade.

### Flaky rolls preparation

Flaky rolls were prepared by designing three different wheat/black rice flour combinations with the following ratios: 100:0 (control), 95:5 (5BR), 90:10 (10BR), and 80:20 (20BR), shown in Table 4. First, margarine and sugar powder were mixed (Electrolux EHM3407) until the mixture was fluffy. Next, the wheat flour, black rice flour, rice protein, cornflour, lecithin, salt, and deionized water were added to the mixture. All ingredients were mixed completely to form a smooth flowing batter. The dough underwent further analysis to determine the pasting properties and rheological characteristics. For the flaky roll preparation, the circular metal pan was preheated to 150°C. Next, a spoonful (15 g) of the batter was spread on the pan. The lid was closed and followed by baking for 30 s. The flaky roll was folded one-quarter on each side, then rolled around a flat rod to produce a flaky roll (baking temperature was 180°C for 10 min).

**Table 4:** Flaky egg-free rolls formulation incorporated with rice protein and different proportions of black rice flour.

**Components (g)**	**Control**	**5BR**	**10 BR**	**20 BR**
Wheat flour	100	95	90	80
Black rice flour	0	5	10	20
Margarine	45.0	45.0	45.0	45.0
Sugar powder	26.7	26.7	26.7	26.7
Rice protein	10.0	10.0	10.0	10.0
Corn flour	1.2	1.2	1.2	1.2
Lecithin	0.1	0.1	0.1	0.1
Salt	0.2	0.2	0.2	0.2
Deionized water	23	23	23	23

BR: black rice, 5, 10, 20: 5%, 10%, 20%.

### Pasting properties

The Rapid Visco Analyzer (Model RVA-3D, Newport Scientific, Sydney, Australia) was used to examine the pasting properties of flour samples with 3 replicates (35). A 3-g amount of flour sample (13% moisture basis) and distilled water (with flour: water ratio of 1:7) were prepared in the test canister and continued to enter a heating-cooling cycle. The starch slurry was equilibrated (50°C, 1 min) at the beginning, then heated to 95°C at 5°C/min, held for 5 min, and continued by cooling at 50°C (5°C/min), then the temperature was controlled at 50°C for 2 min. The viscoamylograph clarified the pasting profiles of the sample as well as the peak viscosity, breakdown, setback, and final viscosity, from the diagram of Rapid Visco Analyzer curves. Three replication tests of each sample were carried out.

### Farinographic characteristics

The *Brabender farinograph* (Brabender farinograph-E, Brabender, Duisburg, Germany) was used to investigate the mixing properties of dough corresponding to the standard method of the American Association for Clinical Chemistry (AACC) 54-21 (2000). Wheat flour with different proportions of black rice flour at 0, 5, 10, and 20% was formulated before analyzing and with the following modifications: (1) weighting the formulated flour at 100 g and pre-mixing for 5 min; (2) adjusting the water addition to the 500 Brabender units (BU) line; and (3) agitating until the graphs of farinograph significantly changed and prolonged to 12 min. Water absorption, arrival time, departure time, stability, peak time, tolerance index, and valorimeter unit were recorded. Water absorption was defined as the percentage of water addition absorbed by the dough to the 500 BU line. Arrival time was defined as the time in minutes measured from the addition of water to the point of the curve band at the 500 BU line. Departure time was defined as the time in minutes measured from the peak of the curve band departing to the 500 BU line. Stability was defined as the difference between arrival time and departure time. Peak time was defined as the time in minutes to the peak of the curved band. The tolerance index was defined as the BU unit difference from the peak of the curve band to another curve band after 12 min. The valorimeter value measured by the farinograph scale was defined as the quality of the dough gluten.

### Extensographic properties

The extensible properties of formulated samples were investigated by using the Brabender extensograph (Brabender extensograph-E, Brabender, Duisburg, Germany) by referring to the standard method of the AACC 54-10 (2000). For the farinograph measurements, the sample was cut into two parts, every part is 150 g. Next, the sample was passed through the balling and mold unit of the extensograph. Stretching the sample every time after 45 min of resting in the fermentation cabinet. Balling and molding operations were repeated after fermentation. The test was carried out in triplicate. The results are expressed as the maximum resistance (BU) to the extension. The extensibility (E) is described as the distance traveled by the recorder paper from the moment the hook touches the test piece until the rupture of the test piece.

### Nutritional composition analysis

The approximate nutritional composition of flaky rolls supplemented with black rice flour, such as the moisture, total sugar, crude protein, crude lipid, total dietary fiber (TDF), soluble dietary fiber (SDF), insoluble dietary fiber (IDF), ash, free sugar, and cholesterol content were studied in 3 replicates and corresponded to the official methods of AOAC 935.39. The moisture content was evaluated by the hot-air oven method; ash content was analyzed by the method of incinerating samples in a muffle furnace at 550–600°C; crude protein content was studied by the Kjeldahl method or Kjeldahl digestion method with conversion factor of 5.7, suitable for wheat; fat content was measured by the acid hydrolysis method; cholesterol content was tested by the digitonin method, and fiber content was studied by the enzymatic–gravimetric method.

### Characteristics and texture analysis

A vernier caliper was used to measure the length (cm), width (cm), and thickness (cm) of the flaky roll supplemented with black rice flour. The other textural properties, such as hardness (N), fracturability (mm), springiness (mm), cohesiveness, and adhesiveness (N.S), were analyzed by a 3-point bend rig (HDP/3 PB) which was equipped with a 5-kg load cell, and heavy-duty platform of the TAXTPlus texture analyzer (Stable Micro Systems, UK). The test speed was set at 3.0 mm/s and the trigger force was set automatically at 50 g. The hardness value represents the maximum force applied, while the fracturability value exhibited the distance at the point of break (13).

### Color analysis

The color of the flaky roll samples was analyzed by the Color Meter ZE-2000 (Nippon Denshku Industries, Tokyo). L^*^ (lightness measurement), a^*^ (greenness-redness value), and b^*^ (blueness-yellowness value) value was studied to determine the quality changes. Calibration of the instrument involved using a standard black-and-white ceramic tile before measurement. Color measurements were carried out at room temperature in triplicate.

### Sensory evaluation

In total, 31 panelists (16 females, and 15 males; age range from 20 to 30 years) from the Department of Food and Nutrition, Providence University, were randomly selected to participate in the sensory evaluation session. The evaluation was carried out in the sensory laboratory at room temperature and strictly followed the GB/T 20980-2007 and ISO 4121 criteria to evaluate the sensory characteristics of flaky roll supplemented with black rice flour in terms of appearance, aroma, taste, texture, and total acceptability. The panelists were required to attend the tutorial and training classes (6 h) to be able to identify and comprehend the rating scales for each attribute before testing. The flaky roll samples for each formulation were cut into a size of 5.0 cm^2^. Samples were selected randomly and placed on white disposable polyform plates marked with a random 3-digit number. All samples were freshly made, and covered with food wrap until testing. The flaky rolls were evaluated by quantitative descriptive analysis involving a 6-point interval scale with scores from 0 to 5 for each attribute, 0 indicating no value and 5 indicating extremely strong value (13).

### Determination of total phenolic content (TPC)

The total phenolic content of the sample was studied by the Folin-Ciocalteu method (36). First, the sample was extracted with 80% methanol. Next, 1 mL extract was added to 10 mL distilled water in a 25-mL volumetric flask. Double distilled water (ddH_2_O) was used as a blank reagent. Then 0.5 mL Folin-Ciocalteu phenol reagent and 5 mL of 5% sodium carbonate (Na_2_CO_3_) solution were added to the mixture solution and mixed thoroughly. The mixtures were diluted with distilled water to 25 mL and allowed to stand for 60 min. Wavelength 750 nm was used to measure the absorbance vs. the blank. Total polyphenol content was calculated by comparing with a standard curve of gallic acid, and the results are expressed as milligram gallic acid equivalent (GAE) per g dry weight (mg GAE g^−1^ DW).

### Determination of total anthocyanins

Total anthocyanin content was determined as described (37) with some modifications. A 500-mg amount of flaky roll samples was placed into a 15-mL Falcon™ tube, then 10 mL acidified methanol with a ratio of 85% methanol: 15% 1 N HCl, was added for extraction. The mixture was homogenized for 30 s by vortex mixing, followed by 30 min of continuous stirring at 200 × g on an orbital shaker. Next, the sample mixture was centrifuged at 7,600 × g for 25 min to collect the supernatant. The absorbance was measured at wavelength 535 nm. Total anthocyanin was expressed as cyanidin-3-*O*-glucoside (CGE) equivalent, and calculated by the formula:

C = (A/ε) × (vol/1000) × MW × (1/sample wt) × 10^6^

Where C = concentration of total anthocyanin (mg/100g); A = absorbance reading at 535 nm; ε = molar absorptivity (cyanidin-3-*O*-glucoside =25,965 cm^−1^ M^−1^); vol = total volume of anthocyanin extract; MW = molecular weight of cyanidin-3-*O*-glucoside (449.2).

### Determination of total antioxidant capacity by 2,2-diphenyl-1-picrylhydrazyl (DPPH)

The free radical scavenging activity of the sample and the standard was evaluated by the free radical scavenging effect of the stable DPPH free radical activity (36). Gallic acid was used as the standard solution, and 0.1 mM DPPH was diluted in methanol. A 2-mL amount of 0.1 mM DPPH was mixed with a 1 mL sample solution or standard solution. These mixtures were kept in the dark for 30 min and the absorbance was measured at a wavelength of 518 nm. The measurements were tested in triplicate.

### Oxygen radical absorbance capacity (ORAC)

The ORAC analysis method used, with fluorescein (FL) as the “fluorescent probe,” was as described (38–40) with modifications. The 96-well microplates were used, and the absorbance was read at wavelengths 485 nm (excitation) and 530 nm (emission). The reaction was carried out at 37°C as the reaction was started by thermal decomposition of 2,2-azobis(2-amidinopropane) dihydrochloride (AAPH) in 75 mM phosphate buffer (pH 7.0). In brief, in each of the 96 -wells was placed 50 μL of 78 nM FL and 50 μL of sample; the blank was the PBS, and 20 μM Trolox was the standard; then 25 μL of 221 mM AAPH was added. To avoid variations in measurement among wells due to the low conductivity of the 96-wells plates, plates were heated up to a temperature of 37°C for 15 min before adding the AAPH. The fluorescence was measured as the relative fluorescence intensity (FI%), measurements were carried out every 5 min until the value was <5% of the value of the initial reading. Each analysis and measurement were taken in triplicate.

### *In vitro* starch digestibility

The *in vitro* starch digestibility of the flaky rolls sample was examined as described (41) with slight modifications. A 200-mg amount of the sample was mixed with 15 mL of 0.2 M sodium acetate buffer (pH 5.2). Next, following by adding 10 mL of the formerly prepared enzyme solution (porcine pancreatic α-amylase [290 U/mL] and glucoamylase [15 U/mL]). The mixture was shaken in a water bath with a temperature of 37°C at 160 revolutions/min. A 0.50-mL amount of the sample solution was collected at different times (0, 20, 40, 60, 80, 100, 120, and 180 min), and the enzymes were inactivated by added in 4.5 mL of ethanol. Glucose content (mg/g) was analyzed by the 3.5-dinitrosalicylic acid (DNS) method.

### Determination of free asparagine content

Free asparagine content was measured as described (29) with modification. A 1-g amount of the test flaky roll sample was extracted with 20 mL of 10 mM formic acid solution and vortexed mixing for 3 min. Carrez I and II solutions were added to the mixture solution to clarify it. The mixture was centrifuged at 15,000 g for 10 min, and the supernatants were stored at −80°C. All extractions were accomplished in triplicate for each sample. The supernatant collected was diluted with an equal volume of acetonitrile and centrifuged at 15,000 g for 10 min. Then, the supernatant was passed through a 0.45-μm nylon filter and collected in a vial before further analysis. The Waters Acquity UPLC system attached to a triple quadrupole detector was applied to investigate the free asparagine content of the sample. Thermo Scientific Syncronis HILIC column (100 × 2.1 mm × 1.7 μm) with a gradient mixture of Solvent A (5 mM ammonium formate in water with formic acid), and Solvent B (5 mM ammonium formate in water: acetonitrile (v/v 1:9) with formic acid) as the mobile phase at a flow rate of 0.7 mL/min at 40°C. The mobile phase gradient was programmed to start with 0% Solvent A, then gradually rose to 80% in 8 min and held for 5 min, then declined regularly to the initial conditions (0% Solvent A) in 2 min and held for 10 min. The electrospray source parameter was as follows: capillary voltage of 3.5 kV, cone voltage of 20 V, extractor voltage of 3 V, source temperature of 120°C, desolvation temperature of 370°C, and desolvation gas (nitrogen) with flow of 900 L/h. Calibration curves were built, and quantifications were from 0.05 to 3.0 mg/L. Theanine as a standard for both working standards and extracts. The results are expressed as mg per kg.

### Determination of acrylamide content

A 1-g amount of ground flaky roll sample was extracted with 20 mL of 10 mM formic acid in 20 mL water and vortexed mixing for 3 min. The extraction and preparation method were mentioned in section determination of free asparagine content. The supernatant collected from the extraction was passed through a preconditioned Oasis MCX solid-phase extraction cartridge, and the pure extract was analyzed by using LC-MS/MS. The extracts for acrylamide were studied by the Waters Acquity H Class UPLC system (Millford, MA) attached to a TQ detector with electrospray ionization operated in a positive mode. The chromatographic separations were performed at a flow rate of 0.5 mL/min on a Thermo Scientific Hypercarb column (100 × 2.1 mm × 3 μm) with the formic acid solution as the mobile phase for 15 min of the isocratic elution. The column was equilibrated at 50°C, while the electrospray source was settings with 2.00 kV of capillary voltage, 23 V of cone voltage, 4 V of extractor voltage, and 120°C of sources temperature, 400°C of desolvation temperature, and desolvation gas (nitrogen) flow 900 L/h. Calibration curves were built, and quantifications were from 1 to 40 ng/mL (1, 2, 5, 10, 20, 40 ng/mL). The results are expressed as μg per kg.

### Determination of HMF and furfural

HMF and furfural content was determined as described (42, 43) with modifications. A 500-mg amount of ground sample was suspended in 5 mL deionized water clarified with 0.25 mL potassium ferrocyanide (15% w/v) and 0.25 mL zinc acetate (30% w/v) solutions in a centrifuged tube. The mixture was centrifuged at 4,500 rpm for 15 min at 5°C. The supernatant was collected and filtered by using a 0.45-μm syringe filter before analysis by HPLC (Shimadzu, Kyoto, Japan). The Synergy 4 μm Hydro-RP 80A, 250 x 4.6 mm (Phenomenex) was used as the column. The mobile phase was a mixture of acetonitrile in water (5% v/v) at a flow rate of 1 mL/min under isocratic conditions for 20 min. The UV detector was set at 280 nm, and HMF was quantified by using the external standard method within the concentration range of 0.025–75 mg/L. All the analyses were performed in triplicate and the results are expressed as mg/kg samples.

### Determination of dicarbonyl compounds, glyoxal (GO), and methylglyoxal (MGO)

In this study, GO and MGO contents were analyzed correspondingly to quinoxalines (Q) and 2-methylquinoxaline (2-MQ) content, respectively, by using a reverse-phase high-performance liquid chromatography (RP-HPLC) procedure attached to UV detection at wavelength 315 nm (44). An LC system (Kyoto, Japan) equipped with an ACEC18 column (5 μm, 250 × 4.6 mm, Advanced Chromatography Technologies, Aberdeen, UK), low-pressure gradient former, pump, and a DAD detector was used. Isocratic elution was accomplished at a mixture of 0.5% (v/v) acetic acid in water and methanol (40:60, v/v) with a detection wavelength of 320 nm. Standard stock solutions of Q and 2-MQ were prepared in ultrapure water to a concentration of 5 mg/mL.

### Statistical analysis

The data are reported as mean ± standard deviation (SD) of triplicate independent experiments. All data were studied by single-factor ANOVA (Turkey test) to determine significant differences at *P* < 0.05. A Microcal Origin program version 2021 (Origin Lab Corp., Northampton, MA) was used.

## Data availability statement

The original contributions presented in the study are included in the article/supplementary material, further inquiries can be directed to the corresponding authors.

## Author contributions

P-HL: conceptualization, investigation, writing—original draft, visualization, writing—reviewing and editing, investigation, resources, reviewing, and methodology. W-CL and P-HL: methodology, resources, and reviewing. W-CL: methodology and resources. Y-TC and P-HL: conceptualization, writing—reviewing and editing, supervision, and project administration. Y-TC, Y-JC, and P-HL: conceptualization, resources, writing—original draft, reviewing and editing, supervision, and project administration. All authors contributed to the article and approved the submitted version.

## Funding

This study was supported by grants provided by the National Science and Technology Council (NSC 110-2622-B-212-001) in Taiwan. This research was also financially supported by Taichung Veterans General Hospital and by Rong Sing Medical Foundation, Taiwan.

## Conflict of interest

The authors declare that the research was conducted in the absence of any commercial or financial relationships that could be construed as a potential conflict of interest.

## Publisher's note

All claims expressed in this article are solely those of the authors and do not necessarily represent those of their affiliated organizations, or those of the publisher, the editors and the reviewers. Any product that may be evaluated in this article, or claim that may be made by its manufacturer, is not guaranteed or endorsed by the publisher.
